# Exploring the User Acceptability and Feasibility of a Clinical Decision Support Tool Designed to Facilitate Timely Diagnosis of New-Onset Type 1 Diabetes in Children: Qualitative Interview Study Among General Practitioners

**DOI:** 10.2196/60411

**Published:** 2024-09-23

**Authors:** Chiara Beccia, Barbara Hunter, Jo-Anne Manski-Nankervis, Mary White

**Affiliations:** 1 Department of General Practice and Primary Care The University of Melbourne Melbourne Australia; 2 National Health and Medical Research Council Centre for Research Excellence in Digital Technology to Transform Chronic Disease Outcomes Melbourne Australia; 3 Primary Care and Family Medicine Lee Kong Chian School of Medicine Nanyang Technological University Singapore Singapore; 4 Royal Children's Hospital Melbourne Australia; 5 Melbourne School of Population and Global Health The University of Melbourne Melbourne Australia; 6 Health Services and Economics Research Unit Murdoch Children's Research Institute Melbourne Australia

**Keywords:** type 1 diabetes, digital health innovation, clinical decision support tool, diabetes, acceptability, feasibility, diagnosis, child, children, youth, qualitative and simulation study, hospital, diabetic ketoacidosis, diagnostic delay, Australian, Australia, video recorded, audio recorded, screen recorded, video, videos, patient, patients

## Abstract

**Background:**

Up to half of the children with new-onset type 1 diabetes present to the hospital with diabetic ketoacidosis, a life-threatening condition that can develop because of diagnostic delay. Three-quarters of Australian children visit their general practitioner (GP) the week before presenting to the hospital with diabetic ketoacidosis. Our prototype, DIRECT-T1DM (Decision-Support for Integrated, Real-Time Evaluation and Clinical Treatment of Type 1 Diabetes Mellitus), is an electronic clinical decision support tool that promotes immediate point-of-care testing in general practice to confirm the suspicion of diabetes. This avoids laboratory testing, which has been documented internationally as a cause of diagnostic delay.

**Objective:**

In this investigation, we aimed to pilot and assess the feasibility and acceptability of our prototype to GP end users. We also explored the challenges of diagnosing type 1 diabetes in the Australian general practice context.

**Methods:**

In total, 4 GPs, a pediatric endocrinologist, and a PhD candidate were involved in conceptualizing the DIRECT-T1DM prototype, which was developed at the Department of General Practice and Primary Care at the University of Melbourne. Furthermore, 6 GPs were recruited via convenience sampling to evaluate the tool. The study involved 3 phases: a presimulation interview, simulated clinical scenarios, and a postsimulation interview. The interview guide was developed using the Consolidated Framework for Implementation Research (CFIR) as a guide. All phases of the study were video, audio, and screen recorded. Audio recordings were transcribed by the investigating team. Analysis was carried out using CFIR as the underlying framework.

**Results:**

Major themes were identified among three domains and 7 constructs of the CFIR: (1) *outer setting—*time pressure, difficulty in diagnosing pediatric type 1 diabetes, and secondary care considerations influenced GPs’ needs regarding DIRECT-T1DM; (2) *inner setting—*DIRECT-T1DM fits within existing workflows, it has a high relative priority due to its importance in patient safety, and GPs exhibited high tension for change; and (3) *innovation*—design recommendations included altering coloring to reflect urgency, font style and bolding, specific language, information and guidelines, and inclusion of patient information sheets.

**Conclusions:**

End-user acceptability of DIRECT-T1DM was high. This was largely due to its implications for patient safety and its “real-time” nature. DIRECT-T1DM may assist in appropriate management of children with new-onset diabetes, which is an uncommon event in general practice, through safety netting.

## Introduction

### Type 1 Diabetes and Diabetic Ketoacidosis

Diabetic ketoacidosis (DKA) is a life-threatening emergency that can occur in up to half of pediatric patients at the diagnosis of type 1 diabetes (T1D) [1]. DKA occurs due to absolute insulin deficiency, a state of metabolic deterioration that can be a consequence of undiagnosed or untreated T1D [2]. A single episode of moderate DKA confers the risk for cognitive impairment in a child’s critical developmental years [3]. It also confers the risk for coma and death [2]. An episode of DKA is also a traumatic event for both the patient and their caregivers, who report higher levels of posttraumatic stress and anxiety following a DKA admission [4].

Diagnosis of T1D before the point at which the child reaches this critically unwell state is challenging. Early symptoms are nonspecific and can often be attributed to more common childhood illnesses, such as an upper respiratory tract infection, or to general childhood growth and development [5]. It also requires a level of public awareness of the signs and symptoms of T1D to facilitate timely presentation to either a general practitioner (GP) or tertiary care to receive life-saving insulin treatment [5].

### Diagnostic Delay Exacerbates DKA Frequency and Severity

Timely diagnosis and treatment are critical, as a diagnostic delay of even 24 hours following symptom recognition confers a 2-fold risk for the development of moderate to severe DKA [6,7]. It has been identified that three-quarters of Australian children with T1D presented to their GP the week before presenting to the hospital [8,9]. The standard guidelines recommend that GPs conduct on-site point-of-care testing and immediately refer a child with suspected T1D to the hospital for a specialist review [10,11]. Diagnostic delay following a visit with a GP can occur due to the provision of an alternate diagnosis [7] and can be compounded by waiting to receive pathology test results before referral to confirm suspicions of T1D [9].

### Our Intervention: Decision-Support for Integrated, Real-Time Evaluation and Clinical Treatment of Type 1 Diabetes Mellitus

Hospital audits examining pathology test practices among GPs demonstrate that in some children, GPs have correctly identified T1D as a potential diagnosis but have elected to request pathology tests instead of direct referral for specialist care. In an Australian setting, it has been identified that 56% of referral delay can be accounted for due to awaiting pathology test results [9]. Electronic clinical decision support tools have shown promise in serving to promote patient safety, leading to changes in clinician behavior that are guideline based and prevent adverse clinical outcomes [12].

In response to diagnostic delay arising from the wait associated with receiving confirmatory pathology results, we have devised DIRECT-T1DM (Decision-Support for Integrated, Real-Time Evaluation and Clinical Treatment of Type 1 Diabetes Mellitus). DIRECT-T1DM is an electronic clinical decision support tool, which provides a real-time alert triggered by the request for a diabetes-related pathology test in a child aged >18 years without an established diabetes diagnosis. The alert advises GPs suspecting T1D as a possibility to consider point-of-care testing instead of venous blood tests in a laboratory, facilitating the patient’s timely referral and safety.

DIRECT-T1DM was designed to work within Future Health Today (FHT), a clinical decision support system (CDSS) that has been developed by the University of Melbourne’s Department of General Practice and Primary Care. FHT is installed in select general practice clinics across Victoria [13] and is compatible with Best Practice and Medical Director, the electronic medical record (EMR) software used by GPs to manage patient care [13]. FHT currently works as an after-the-fact analysis by reading EMRs on a centralized server within the practice overnight, applying coded algorithms to identify patients who may benefit from further review. An onscreen pop-up activates when the patient file is opened (usually when the patient returns for a visit) and displays recommendations for patient care. In contrast, DIRECT-T1DM is designed to activate in real time using a combination of information stored in the FHT server and information entered into the EMR during a consultation.

**Figure 1:**
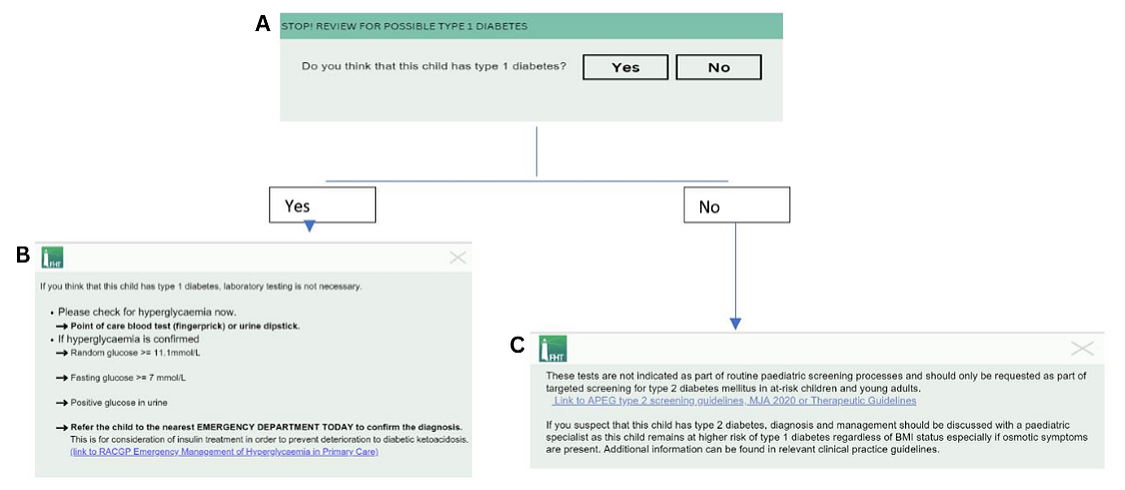
DIRECT-T1DM (Decision-Support for Integrated, Real-Time Evaluation and Clinical Treatment of Type 1 Diabetes Mellitus) clinical decision support pop-ups. This figure is a flowchart of the alert that appears on the computer screen when DIRECT-T1DM is triggered. The initial alert (A) reads “STOP! Review for possible type 1 diabetes” in a green header, with a gray textbox below it asking, “Do you think this child has type 1 diabetes?” The general practitioner (GP) has the option to either select yes or no. When the GP selects yes (B), a new alert comes up describing the clinical action to take. When the GP selects no (C), a different alert appears.

The DIRECT-T1DM pop-up has 3 components. The initial part of the pop-up (Figure 1) asks the question: “Do you think that this child has new onset type 1 diabetes?” Depending on the response, clinical advice is provided to the GP regarding the recommended next steps.

Acceptability of electronic clinical decision support tools by GPs is a strong facilitator of their use within the community [14]. Elements that need to be designed effectively to increase usability and acceptability include user-friendliness and compatibility with workload and workflow, and decreased relevance of information are barriers to the use of clinical decision support [14]. Ascertaining whether these elements are acceptable to GPs is therefore an important aspect of our implementation process.

The aims of this investigation were to assess the acceptability of the DIRECT-T1DM software program by end users, by testing the program in a simulated clinical environment, and to explore the additional factors influencing T1D diagnosis that could be used to optimize DIRECT-T1DM.

## Methods

### Overview

We conducted qualitative interviews with GPs to evaluate the acceptability of the DIRECT-T1DM software program. Content analysis underpinned our methodological orientation. The qualitative interviews were conducted in a simulated environment in 3 steps. First, a presimulation interview was conducted to understand the prior experience of the GPs. Next, simulated clinical scenarios were tested so that GPs could evaluate the software in the context of which they would use it. Finally, a post simulation interview took place to evaluate the tool and provide feedback. The COREQ (Consolidated Criteria for Reporting Qualitative Research) framework was used and can be found in Multimedia Appendix 1.

### Research Team and Reflexivity

CB conducted the interviews with all participants. CB is a female PhD candidate, and the results of these interviews will form part of her thesis. She has prior experience in conducting qualitative interviews with health professionals and works with GPs frequently in her research. She had preexisting professional relationships with 3 out of the 6 GPs before beginning the interview process. One GP interviewee has collaborated with CB in prior research in the same field of DKA prevention and, therefore, may have had prior knowledge of existing guidelines and reasoning for the implementation of DIRECT-T1DM. Steps were taken in the design of the interview guide (Multimedia Appendix 2) to manage any preexisting relationships with the participants by delineating clear roles during the interview and by an initial briefing and debriefing about the research (refer to the before and after interview schedules). The influences of these participants’ responses were considered during analysis and are acknowledged in the *Strengths and Weaknesses of This Study* section. BH, JAMN, and MW were CB’s PhD supervisors, who collaborated in the development of DIRECT-T1DM, construction and editing of the interview guides, recruitment of the participants, discussion, and provision of feedback on interviews as they were conducted. They also provided feedback on the results and analyses that emerged from the study and edited this publication. BH is a qualitative and implementation researcher, JAMN is an academic GP, and MW is a pediatric endocrinologist and health services researcher.

### Software Development

Business requirements and algorithm design documentation for the DIRECT-T1DM prototype were submitted in February 2023 to the FHT technical team at the University of Melbourne. One GP, a pediatric endocrinologist, and a PhD student were the investigators on this project and were involved in the early-stage development of the tool. The tool was then presented to an additional 3 GPs for their feedback on where to improve the design aspect. The design requirements were then passed on to the software developer within the FHT technical team for production of the prototype.

### Research Design: Qualitative Interviews and Simulation

GPs were invited to evaluate DIRECT-T1DM at the Digital Health Validitron SimLab at Melbourne Connect, a University of Melbourne facility designed to conduct user acceptability testing of digital solutions in near-live settings [15]. This involved a presimulation interview, providing them context about the purpose of the investigation and the prototype; the simulation itself, involving subset of possible 5 clinical scenarios where the pop-up would be triggered, facilitating on-the-spot feedback for the tool; and a postsimulation interview discussing their experience using the prototype. Each session lasted approximately 60 minutes.

The presimulation interview included questions surrounding the GP’s experience of practicing, practice location, and an estimation of the proportion of pediatric patients that they would see on an average day. It also involved a discussion around T1DM, whether they had diagnosed it before, and any challenges involved with this. An explanation of the prototype, its purpose, and the simulation were then provided.

The simulation session involved a “think-aloud” protocol [16], where GPs were instructed to be as verbose as possible throughout the clinical scenarios and their interactions with the pop-up. This was done with the intention of collecting on-the-spot feedback about the pop-up tool from each GP. GPs would engage with 2 allocated clinical scenarios and were presented with a third if time permitted. All clinical scenarios were designed with input from GPs. JAMN, a member of the study, contributed to the design of the clinical scenarios, and 3 GPs, independent to the study, were briefed on the study aims and provided brief clinical scenarios for feedback. This feedback was then used to optimize the scenarios to ensure that we could meet our study aims. In total, 2 out of 5 scenarios were related to a child presenting with symptoms suggestive of T1DM, and the remaining 3 scenarios were related to presentations suggestive of type 2 diabetes, polycystic ovary syndrome, or where a family sought to discuss the screening for T1D in the setting of a positive family history. Clinical scenarios were designed to warrant the request of a diabetes-related pathology test, such as HbA_1c_ or fasting blood glucose, thereby triggering DIRECT-T1DM. Clinical scenarios were presented by the interviewing researcher, not by a patient actor. The reasoning for this was three-fold:

1. The intention of the interviews was to assess the acceptability of DIRECT-T1DM, not to test whether GPs were aware of the guidelines. It allowed triggering of the tool so that participants were guaranteed to interact with it, and having the researcher deliver the clinical scenarios allowed GPs to provide instant feedback as they interacted with the tool.
2. Pediatric T1D symptoms before diagnosis are often vague and nonspecific; therefore, a simulated consultation with a patient actor may lead to T1D never being suspected by the GP.
3. If GPs suspected diabetes, they may have elected to complete the point-of-care testing instead of referring for pathology and, therefore, never interact with DIRECT-T1DM.

The postsimulation interview was designed using the Consolidated Framework for Implementation Research (CFIR) [17], evaluating GPs’ appraisal of the tool based on several key domains and settings: inner setting, outer setting, innovation, roles, individual characteristics, and implementation process [17]. CFIR is a commonly used, comprehensive framework used to ascertain key contextual determinants of success or failure of an intervention as well as for appraisal of the implementation process, facilitating optimization of interventions before deployment into the broader community [17].

### Participant Selection and Recruitment

GPs were recruited via email through the personal networks of the investigators as well as through the Victorian Practice Based Research Network [18]. We aimed to recruit GPs of different genders and various levels of experience by ensuring our approach involved practices with an adequate representativeness of junior and senior GPs as well as gender balance. Some nonparticipants were present during the interviews, including staff running the SimLab as well as additional researchers on the investigating team. Participants were made aware of their presence and introduced to the nonparticipants.

### Data Collection

The interview guide designed for all 3 stages of the study was devised, pilot-tested, and optimized by CB and BH. Video, audio, and screen recording were completed using SimLab resources. Brief field notes were collected by the interviewing researcher to reflect on her interviewing technique. Copies of transcripts and summaries of the results of this study were offered to participants upon request. The interviews lasted between 40 and 60 minutes.

### Data Analysis

Analysis was facilitated by NVivo (version 14, release 1.6.1; QSR International). CB initially familiarized herself with the data by transcribing the audio recordings verbatim and reading each transcript line by line. The domains and constructs within CFIR (5 domains and 39 constructs) were transposed into the NVivo codebook. CB coded the data, grouping codes deductively into the constructs within CFIR. No domain or construct within the codebook was removed before initial analysis. Following initial analysis, themes within CFIR constructs were inductively generated. Following this, domains and constructs that did not pertain to our simulation study were removed. The decision made for this involved charting the existing codes to determine whether they were major or minor themes and whether they could be assigned to a different CFIR domain. The domains and their related constructs that were removed were individual characteristics and roles. This is because they did not map to the aims of our investigation, which heavily involved appraising the “innovation” domain. It was identified that CFIR constructs within domains did not exist in isolation when appraising our tool; thus, visualization of how the themes link from one to the next was undertaken, as demonstrated by Sarkies et al [19]. No double coding was conducted on the data, and all data analyses were conducted by the primary author CB.

### Ethical Considerations

The ethics application for this project was approved by the Human Research Ethics Committee at the University of Melbourne (ethics ID: 2023-25185-45082-4). Informed written consent was obtained for each of the participant involved in this study. Transcripts were deidentified, and published results attribute responses to a deidentified code representing each participant. GPs were compensated with an Aus $200 (US $1=Aus $1.48) VISA Gift Card for their participation in the study. No participants withdrew consent, and no interviews were repeated.

## Results

**Figure 2:**
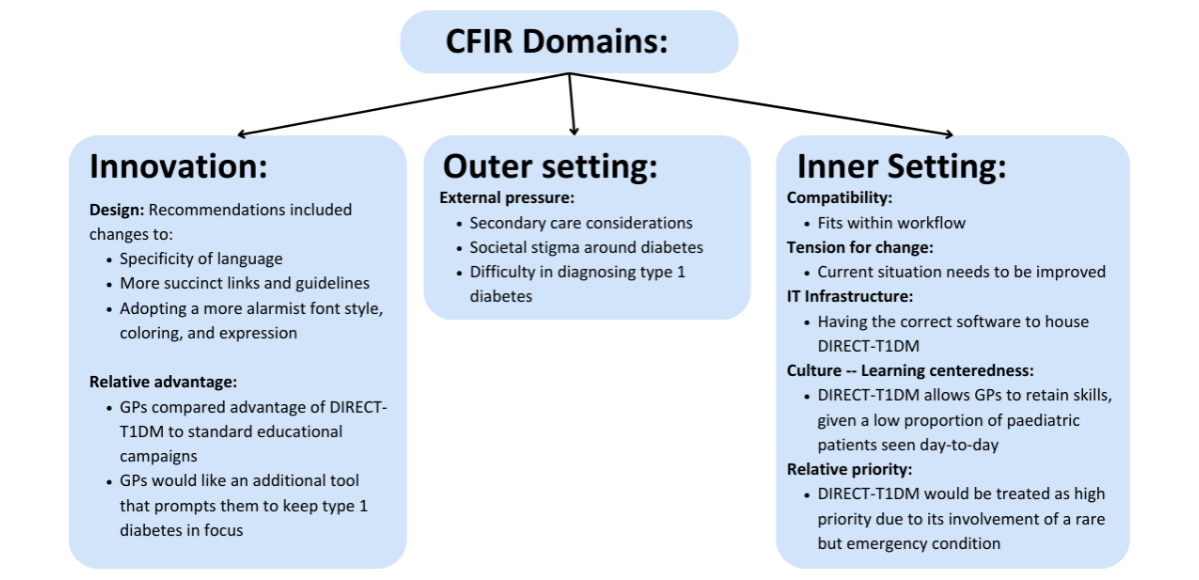
Summary of the key results across all Consolidated Framework for Implementation Research (CFIR) domains. This figure describes the 3 CFIR domains that were included in this study: innovation domain, outer setting, and inner setting. This is a flowchart that starts with the words “CFIR Domains” at the top, which splinters off into innovation, outer setting, and inner setting categories. In each category, there is a summary of important points derived from relevant CFIR constructs. DIRECT-T1DM: Decision-Support for Integrated, Real-Time Evaluation and Clinical Treatment of Type 1 Diabetes Mellitus; GP: general practitioner.

Figure 2 outlines a summary of the key results across 3 CFIR domains and 7 constructs. The results summarized here are expanded upon in subsequent sections.

### Participants

In total, 6 GPs were recruited to take part in the simulation study. Participants were diverse in gender, years of GP experience, and exposure to CDSS. Two were academic registrars completing their general practice specialty training with <2 years of experience, 2 had 3 to 5 years of experience, and 2 had >30 years of experience working as a GP. Three had experience of using FHT in their practice. All were familiar with the Best Practice medical software. Table 1 summarizes the participant demographic information.

Of the 6 participants interviewed, only 1 (16%) GP had previously diagnosed a child with new-onset T1D before these interviews. As such, the scenarios were not instances the participants had encountered in practice, and their initial responses were based on how they believed they would have responded in practice. Participants were also explicitly asked to interact with the scenarios in a manner that would trigger the prompt (potentially not following best clinical practice), to explore the acceptability and feasibility of the tool. Some GPs reflected on what they would have done in response to each clinical scenario in practice and whether T1D would have crossed their mind in this case. One GP stated that preexisting awareness of the investigation before engaging in the interviews would skew a participants’ response to the scenarios, especially given the scarcity of T1D presentations in practice. The think-aloud protocol was beneficial for on-the-spot feedback, appraisal, and reflection regarding DIRECT-T1DM, meeting the aims of our investigation. However, it should be noted that the burden on GP participants was increased, when compared to “near-live” scenarios or real life:

I didn’t read too much about [T1D] or anything, because sometimes I think it’s better walking into something like this a bit fresh...

GP5

Yeah, I think as a participant, you probably overthink things and second guess yourself more than you would do in real life. I think even just taking your history felt really painful just now compared to real life. But that’s, I guess that’s part of the process. I think though as far as participating in something it’s very easy.

GP3

**Table 1:** Participant demographic information. This table describes the GPs^a^ interviewed, their gender, years practicing, familiarity with Best Practice, FHT^b^, and whether they had diagnosed pediatric T1D^c^ in the past.

GP	Gender	Years practicing	Familiarity with Best Practice	Uses FHT in practice	Diagnosed pediatric T1D (number of patients diagnosed)
GP1	Male	>30	Yes	No	No
GP2	Male	<2	Yes	No	No
GP3	Female	<2	Yes	No	No
GP4	Male	3-5	Yes	Yes	No
GP5	Female	3-5	Yes	Yes	No
GP6	Female	>30	Yes	Yes	Yes (n=1, 16%)

^a^GP: general practitioner.

^b^FHT: Future Health Today.

^c^T1D: type 1 diabetes.

### Innovation Domain

#### Acceptability of DIRECT-T1DM

The CFIR construct we aimed to assess in our interviews primarily involved the innovation domain, appraising DIRECT-T1DM itself, its acceptability, and proof-of-concept. We found that DIRECT-T1DM was highly acceptable to GPs. This is because DIRECT-T1DM appeared because of an action the GP had taken during the consultation. The GPs did not anticipate encountering this tool often, as pediatric patients do not constitute a sizeable proportion of their consultations, and of those pediatric consultations, they do not anticipate requesting many pathology tests. GPs recognized the need for this tool, stating its importance as a safety measure, to prevent potentially fatal sequelae. Design recommendations were provided to optimize DIRECT-T1DM by addressing specific GP needs at the point of care, enhancing compatibility with GP workflow and workload, and alleviating pressures arising from both the inner and outer settings. Major constructs that emerged from the innovation domain were design and relative advantage. A summary of the key results across all CFIR domains can be found in Figure 2.

#### Construct 1: Design

In CFIR, design refers to the degree to which the intervention is well packaged and presented [17]. Major themes within this construct were surrounding the language, structure, available links, coloring, and emphasis within DIRECT-T1DM.

##### Language and Content

###### Overview

Table 2 illustrates the key responses to DIRECT-T1DM as they pertain to language and content.

**Table 2:** Key responses to DIRECT-T1DM^a^ as they pertain to the language and content used within each of the pop-ups.

Section of DIRECT-T1DM and key feedback			Representative quotes
**Initial question: Do you think that this child has T1D^b^? (Figure 1A)**.			
	Pose the question of T1D as a *possibility* not a certainty	“It’s pretty obvious in this case, but what if it wasn’t so obvious? What if I said I’d been tired and lost a few kilos, not sure, but nothing is acute though, so it could be just anything else but diabetes, then you do a sugar. It’d be, I wouldn’t say I think he had it, I think, is it possible? Yeah, it’s possible. So that might prompt me to do the fingerprick test.” [GP^c^6] “It’s almost as if the pop up needs to say, instead of saying, do you think this child has? Because that’s kind of what’s most likely. Yeah, it could be like, is there a chance this child has type one? Something a bit more, not on the balance of probabilities, but more on, you don’t want to miss type 1, so is there a chance this child has type 1?” [GP5]	
	GPs appreciated the succinctness of the initial alert	“It’s good. It’s a simple question. Because it’s giving you a differential that you may or may not have.” [GP3] “That’s why it’s good to be reminded. Hang on, stop, clinical point here. We want you to you know it’s really good to check the sugars now because this person at high risk who might need urgent action.” [GP6]	
**Instructions that appear after you select yes (Figure 1B**).			
	Information was necessary, actionable, and specific enough for the point of care	“It’s really clear—you know what’s normal and what’s abnormal and when the child needs to go to emergency and how urgently.” [GP3]	
	Reference ranges were helpful	“I like the fact that it reminds you of the reference ranges for [blood glucose levels].” [GP2]	
	Make the clinical action “stand out”	“I think I’d change it to make [a point of care blood test] really stand out. Yeah. Yeah, maybe even like, a box like. ‘Please check your pinprick sugar and urine ketones.’ I would make those stand out, like bang and bang. That’s the important information. That’s the action. This is what to do with information.” [GP6]	
**Instructions that appear after you select no (Figure 1C) **.			
	Specific phrasing: “routine paediatric screening” was confusing	“[Reading DIRECT-T1DM recommendation] ‘These tests are not indicated as part of routine paediatric screening.’ I’m a little confused by that.” [GP1] “I guess it’s a bit confusing because it’s saying you shouldn’t use them as a routine screening. There’s not really any, not really much routine paediatric screening. Yeah, like there’s not bloods that we order on every child. The routine immunization and, you know, once they get to a certain age, cycle screening all that, but I’m not quite sure what that means.” [GP5]	
	Information in this part of the alert was too restrictive for general practice	“I would word it differently. ‘This is only to be requested as part of targeted screening.’ I think this sounds too prescriptive and that it doesn’t... Because that doesn’t take into account experience, what the patient might want, etc, their baseline, you know... So, I suppose, I don’t want that. I would just click out of it and order the test... It would be another annoying, that’d be an annoying pop up for me. Wouldn’t be helpful.” [GP6] “Yes, I think less words... So, if you’re trying to make people more aware of missing type one diabetes, I would just focus on that rather than sort of talking about routine screening.” [GP5]	
	Explain osmotic symptoms	“Is this bit about risk? So regardless of BMI status, especially if osmotic symptoms are present. Osmotic symptoms. Would be more helpful if that was a bit more specific.” [GP2]	

^a^DIRECT-T1DM: Decision-Support for Integrated, Real-Time Evaluation and Clinical Treatment of Type 1 Diabetes Mellitus.

^b^T1D: type 1 diabetes.

^c^GP: general practitioner.

###### Positing T1D as a Possibility Rather Than a Certainty

During the delivery and think-aloud response to the clinical scenarios, it became clear that often, even when “typical” symptoms of T1D were present, the clinical suspicion of T1D was low. Therefore, this indicated that in general, GPs may be requesting diabetes-related pathology as 1 part of a panel of several different tests to ascertain what condition the child has presented with. All GPs participating in our study requested a panel of different pathology tests alongside a blood glucose test when demonstrating what they would do in practice. This led to a design recommendation surrounding an alteration in the language used in the initial pop-up. GPs suggested that in most cases, it was likely that they would be uncertain that the child had T1D, as children may not appear emergently unwell. Consequently, they believed that adjusting the language in the initial question from “Do you think this child has type 1 diabetes?” to “Is it possible that this child has type 1 diabetes?” would change their immediate reaction to DIRECT-T1DM. They emphasized a shared belief that it is important to still interact with DIRECT-T1DM, even in cases where there is a low degree of suspicion of pediatric T1D, and they believed that an alteration in language will prompt them to exclude the possibility of DKA through a point-of-care test before moving on to requesting further pathology.

###### Increasing Language Specificity to Tailor the Tool Optimally for the Point of Care

There was consensus regarding increasing the specificity of the language within DIRECT-T1DM. In sum, time pressure in practice necessitates pop-up alerts with information that is short, sharp, and directly actionable at the point of care. Increasing specificity of language within the tool will also decrease the cognitive load required of the GP to interpret it midconsultation. This is important with our tool, as it is disruptive by design. The initial question “Do you think that this child has type 1 diabetes?” was appraised as fitting within the needs of GPs during that point of the consultation, as it was concise and interrupted their clinical decision-making at an appropriate point in time.

When GPs selected “Yes” to the initial question, they are prompted to complete a point-of-care test instead of pathology testing to prevent deterioration to DKA. GPs agreed that the information provided within this tool was necessary and specific enough for actioning within the time available. The provision of reference ranges and instructions for further management should the child have hyperglycemia was clear and helpful. More emphasis could have been placed on the initial part of the messaging to emphasize the purpose of the point-of-care testing and decrease the cognitive load required to interpret the messaging within this part of DIRECT-T1DM.

When GPs select “No” in response to DIRECT-T1DM’s initial prompt (“Do you think this child has type 1 diabetes?”), GPs were confused by the phrase “not indicated as part of routine paediatric screening processes.” Some pointed out that there are no routine pediatric screening processes in general practice. Others believed that the text was too restrictive and prescriptive for the context of general practice. The consensus recommendation was rephrasing of the text to centralize and emphasize its key purpose. One GP also stated that due to its restrictive nature, it would be a recommendation that would be resented and not used in the community. Several GPs also reported that they would prefer a brief explanation of what osmotic symptoms are or to replace this phrasing with more specific language delineating these symptoms instead.

##### Positioning on Screen, Visual Structure, Links and Guidelines, Font Colors, and Emphasis

###### Overview

Table 3 summarizes the key feedback provided to us regarding positioning on screen, visual structure, links and guidelines, fonts, colors, and emphasis.

**Table 3:** Key responses to the positioning on screen, visual structure, links and guidelines, fonts, colors, and emphasis in each of the pop-ups.

Theme and key feedback			Representative quotes
**Positioning on screen**			
	There is limited time available in the clinic to review pop-ups in their entirety, and some as a result are relegated to a lower priority.	“Just with that, it is just time pressure. So, I’m guilty of it. Like if I’m running behind and the pop up will just appear in the background and I probably won’t look at it, even though I’m I know I’m supposed to.” [GP^a^4] “I just feel sad that I just don’t even notice the Future Health Today ones as much now... and I wish I did, but I just don’t because, as I said, I think there’s so much happening in consultation and it’s just another thing, you know. Yeah, it just that’s one thing I can ignore and pull my energy into the patient.” [GP6]	
	DIRECT-T1DM^b^ has an attention-grabbing central position on screen, which makes them less likely to miss the pop-up. They felt that, due to its implications for patient safety, that this pop-up’s central location is warranted.	“As a registrar, I’m acutely aware of the limitation of just the less experience you have, the more likely you are to miss things. So, if you have something to remind you or to make you think naturally, you’ll remember the differential that you had, and particularly when you’re struck for time, running late, all that, all those factors that make us, rush or forget things and that sort of a safety net for us to remember and to not under- or over-investigate them.” [GP3] “I like that it’s, you know, big bold letters. Stop. Consider type one diabetes. I think it’s just the right level of sort of intrusive. You know it, it should pop up and be in the middle of the screen. It’s appropriate.” [GP2] “It’s also something you could even just show the patient or the parents as well, because if it’s a sort of clinical decision-making thing, a lot of people don’t like the idea of having to go to emergency... So, this I think is helpful that it’s right on the screen there. I use it for drug interactions as well.” [GP5]	
**Visual structure and design**			
	The simpler the design and structure, the easier it is at the point of care.	“...maybe with the first part just have the first line. So please check for hyperglycaemia now and then point of care test and then maybe have another to pop up afterwards...so I think it just breaks it up. That’s just probably me, because I think, just I don’t like to read too much text on my screen.” [GP4]	
**Links and guidelines**			
	Links provided to documents and guidelines should be succinct and focused for the clinical action that is relevant.	“If you select the type 2 or you don’t think that it’s type 1 diabetes, that one I found a little bit more confusing... And that the link didn’t really give me any good specific information on a, you know, a child, a child with suspected type 2 diabetes...” [GP1] “...I think it’s a little bit confusing the way it hooks between the type 2 and type 1 and refers to sort of very long guidelines and things. And I think the focus should just be on diagnosis and screening, like management is a sort of tomorrow problem... You’re probably not even going to not to find the section in the time frame that you have to make a decision...” [GP5]	
	Include patient communication material should a GP have to make a referral to emergency.	“But when you send the hospital, the other pop up which would be useful would be probably a fact sheet for parents as well...What diabetes is, what the management is, why we’re worried about it and why we have to send to the ED.” [GP2]	
**Color, font style, and emphasis**			
	Make the design of the alert more conspicuous.	“Oh, actually I like the colour, it’s calming. But I wonder if red makes it more like...more like a health alert or something, you know. You want it to sort of bang!” [GP6]	

^a^GP: general practitioner.

^b^DIRECT-T1DM: Decision-Support for Integrated, Real-Time Evaluation and Clinical Treatment of Type 1 Diabetes Mellitus.

###### Positioning on Screen

GPs reported that the available time in clinic to review pop-ups while seeing patients is often limited, and as a result, pop-ups that appear may be relegated to lower priority of items they must address during a consultation. However, GPs felt that DIRECT-T1DM’s attention-grabbing central screen position with bold font, which was different from the location of other FHT prompts, assisted them in engaging with the directive in the pop-up, even when this time pressure existed. They felt as though they were less likely to miss this pop-up, even when under pressure to undertake a consultation in a short amount of time or impacted by alert fatigue and could use the alert to also engage with patients about the importance of referral, if required.

###### Visual Structure and Design

GPs indicated that optimizing DIRECT-T1DM to have a more simplistic visual structure will increase their engagement with the tool and contribute significantly to decreasing the cognitive load required to appraise the information held within the tool, allowing them to keep the rest of their attention to the patient during the consultation. All GPs agreed that the clinical recommendations had too many words. Some GPs recommended checkboxes, while others suggested that the information was provided in a two-step structure: (1) complete a point-of-care test now to prevent deterioration into DKA and (2) reference ranges and further action depending on the point-of-care results.

###### Links and Guidelines

GPs who suspected a diagnosis of T1D were directed to the Royal Australian College of General Practitioners “Management of Hyperglycaemic Emergencies” document; GPs would have preferred it if the link immediately directed them to the flowchart for management. The links providing guidance when T1D was not suspected (Management of type 2 diabetes: A handbook for general practice [20] and the Australian Paediatric Endocrine Group type 2 diabetes screening guidelines [21]) were too long—198 and 14 pages, respectively—and did not contain direct and focused instructions that could direct treatment at the point of care. Several GPs suggested also adding a patient information sheet to DIRECT-T1DM to aid in explaining the child’s situation to parents who go to an emergency department (ED) following a visit with the GP.

###### Color, Font Style, and Emphasis

Red coloring was suggested in place of the green color scheme embedded within DIRECT-T1DM due to a desire for this to raise alarm among GPs regarding the safety of the child and the urgency of their situation. A more consistent style of font was desired across all parts of DIRECT-T1DM. Other signifiers of emphasis on our tool were desired, such as exclamation marks, bold font, and underlining.

#### Construct 2: Relative Advantage

##### Overview

Relative advantage refers to the extent to which the innovation is better than existing innovations targeting the same issue or current practice [17]. Two themes emerged when discussing relative advantages with interviewees: (1) access to CDSS compared to GP education or public awareness campaigns and (2) relative advantage in cases where T1D may not be initially recognized by GPs. Textbox 1 summarizes the key responses to DIRECT-T1DM as they relate to relative advantage.

**Key themes related to relative advantage.**

**Education programs alongside Decision-Support for Integrated, Real-Time Evaluation and Clinical Treatment of Type 1 Diabetes Mellitus (DIRECT-T1DM)**

- “I think [DIRECT T1DM’s] one part of the solution. I think probably awareness raising both with the general public and in general practice. Both of those factors can complement each other.” [general practitioner; GP1]

**Risk prediction tools: what do we do when type 1 diabetes (T1D) is too difficult to recognize?**

- “It would be great if we had this artificial [intelligence] system that can [read] through the patient history and detect type one [diabetes] for the children and then just [tell the GP] consider [diabetes] or something.” [GP3]
- “Just trying to think. Like whether it would also be useful and maybe future health today already does this, but like even just weight loss in someone under 18. Like it would almost be good if that could trigger [DIRECT-T1DM] alone.” [GP5]

**Other issues to consider due to the pediatric setting**

- “I think the challenges initially are going to be because they’re a child. So, the younger they are, the less capacity they have to give you a history, and you’re relying on parents or carers for information. I think the other difficulty with children is that they probably present, maybe less typically and perhaps with symptoms that you might not necessarily associate with you know, diabetes, new onset diabetes.” [GP1]

**DIRECT-T1DM reminds you to check for T1D in cases that may appear mild or where suspicion may be low.**

- “Yeah. So, it prompts you to do the point of care testing. Yeah, which a barrier often is time. Yeah, I think, yeah. If I’m honest, it’s the right thing to do. And we should always do it. But if I’m meeting this child the first time, they’re just taking a real long history... maybe it’s easier for me to just order the [test] and the blood test form and send along.” [GP3]
- “Because this guy is, I’m worried about. OK, I’ll probably send to hospital. Do you see what I’m saying? So, it’s more the grey, the grey area thing. So, I think I think it’s a good idea to do it to prompt the GP to do a pinprick at a lower level of suspicion.” [GP6]

##### Education Programs May be Desired Alongside DIRECT-T1DM

Some GPs felt that an awareness campaign or GP education may also be helpful in the prevention of DKA and that these preferences would likely vary by GP. Implementation of DIRECT-T1DM could benefit from pairing with an awareness campaign.

##### DIRECT-T1DM Should Be Used in Conjunction With Other Methods in Cases Where T1D Is Hard to Recognize

GPs were additionally concerned about whether they were keeping pediatric T1D in focus during their day-to-day consultations. Some GPs expressed that this could result in delays that would not be addressed by the DIRECT T1D tool, which focused on potentially inappropriate pathology requests. Many GPs stated that they would like a prompt that would help them consider T1D based on the child’s prior medical history or triggered based on the symptom input at that consult. This way, diabetes is kept in focus and they can complete a fingerprick and direct future management as required. DIRECT-T1DM’s trigger point, following the ordering of diabetes-related pathology, may not catch all children who attend general practice with new-onset T1D.

GPs felt that diagnosis was also made difficult due to the pediatric setting, as children are unable to verbalize their symptoms as well as an adult may be able to, limiting their capacity to give an accurate history. Children may also vary more widely in their presentation, with symptoms less attributable to T1D. GPs attributed the relative obscurity of T1D-related symptoms, combined with the lack of time available in practice as another potential reason why they may elect to order the pathology test externally rather than complete point-of-care testing. Often, symptoms of T1D sit in a “grey area,” and the urgency of a diagnosis may not be apparent, as children may appear well or not any less well than children with other common conditions, such as viral illnesses.

### Inner Setting Domain

#### Overview

The inner setting domain is the setting within which the intervention is implemented [17]. In our case, this would be the general practice clinic. The 5 constructs within the “inner setting domain” predominantly assessed in our interviews were tension for change, relative priority, compatibility, learning centeredness, and IT infrastructure. Table 4 summarizes the responses from GPs to each construct within the inner setting domain.

**Table 4:** Constructs and themes discussed for the inner setting domain.

Construct and key themes			Representative quotes
**Tension for change**			
	All GPs^a^ recognized a need for the current situation to improve.	“You hear about people attending emergency and being diagnosed [with T1D]. So, if someone were to sort of walk into my room with [T1D], I wouldn’t mind a sort of slightly alarmist pop up.” [GP5] “I’d prefer to do some sort of point of care test whether it’s a, you know, a pinprick or a urine, at least to get an idea of where we’re at. Yeah, on the spot rather than doing the pathology. So, I think that recommendation is very reasonable.” [GP1]	
**Relative priority**			
	DIRECT-T1DM^b^ takes high priority because it would appear rarely in practice and its purpose in enhancing patient safety.	“So, I think yeah, based on that because it’s a paediatric age group and cohort then probably wouldn’t, you wouldn’t get it often, so that when you did get it you would take it seriously.” [GP3]	
**Compatibility**			
	DIRECT-T1DM appears at the right time during workflow.	“And I think it comes up at the right time, like when you’re sort of, you know, maybe you’re sort of trying to decide do I order bloods and bring them back or do I actually just need to send them now? Like it’s a good prompt at that time, or you’ve got the file open and everything, yeah.” [GP5]	
**Culture** **—** **learning centeredness**			
	GPs appreciated the retraining in pediatric-related medicine as they recognize that you may deskill as you see fewer pediatric patients	“Like it’s not that often that I’m ordering a sugar test, in a young person, that it would, you’re not going to sort of get pop up fatigue from it. I think it would be generally, especially maybe because we don’t see a huge proportion of paediatric patients [in this clinic]... it’s almost more necessary I think, because you have to think of what is going to affect them differently as to a 60 year old male or whatever, but they’re almost a different species in a lot of ways. So yeah, you kind of deskill a little bit if you’re seeing less of them.” [GP5]	
	Teaching practices have an emphasis on evidence-based, guideline-driven care. DIRECT-T1DM fits within this culture.	“From my experience with talking with other clinicians about this, the people who seem the most interested and accepting of it are property more junior or doctors who have maybe graduated, you know, maybe within the last five to 10 years, I think because they’re a little less fixed in their in their ways and the way they practice, very guideline driven, very evidence driven and sort of. I think would appreciate these tools whereas I think maybe some older clinicians who have like vast amount of personal experience probably might not be as accepting of it.” [GP2] “I think most people would find it helpful, even those that are very experienced, you know, for the most part. Lifelong learners as doctors and really open to changes in guidelines and, you know, being prompted to consider things that may not have. I think teaching practices in particular, and obviously as a registrar, you’re in a teaching practice. So yes, most people are pretty open to it.” [GP3]	
**IT infrastructure**			
	A potential barrier to clinical decision support is convincing clinics to install the software that sits outside the EMR^c^, rather than the clinical decision support tool itself.	“That would be the barrier, yes. Not so much the usefulness of the pop up, but actually getting additional software or software that engages with the EMR and has the potential to have privacy issues. I think other general practices are wary of because of data breach episodes...” [GP1]	

^a^GP: general practitioner.

^b^DIRECT-T1DM: Decision-Support for Integrated, Real-Time Evaluation and Clinical Treatment of Type 1 Diabetes Mellitus.

^c^EMR: electronic medical record.

#### Tension for Change

GPs welcomed the implementation of DIRECT-T1DM, thus recognizing the need for a change to the current practice. Two GPs were also aware of children attending the ED with DKA in part due to delay from pathology test requests, emphasizing the need for the implementation of DIRECT-T1DM because of its benefits to patient safety. The other GPs still recognized a need for change to current practice, even if they were not aware of any specific patient circumstance relating to DKA presentation because of delayed diagnosis of T1D. They valued the introduction of DIRECT-T1DM because of its purpose as a safety mechanism to ensure that patients in urgent need of care receive it in a timely manner.

#### Relative Priority

Despite receiving numerous electronic pop-ups and alerts each day, GPs stated that DIRECT-T1DM would have a higher relative priority when compared to other interventions. This was because they did not anticipate encountering it often, and its purpose in preventing deterioration to DKA heightened the likelihood that they would engage when it does appear.

#### Compatibility

GPs stated that DIRECT-T1DM integrated well with their workflow, as it interrupts their clinical decision-making at the exact right point in time, when they go to press “print” on a pathology test, giving them the chance to reverse their choice, ascertain the safety of continuing with the pathology testing, and move on from there. GPs found the flow of the instructions within DIRECT-T1DM to be compatible with their workflow, as the provision of reference ranges for blood glucose levels enabled them to direct future management on the spot, saving time that would have been spent looking for the appropriate guidelines. As previously stated, when appraising intervention design, the structure and visual aspect of the tool could be improved to decrease the cognitive workload placed upon the GP.

#### Culture: Learning Centeredness

GPs had a positive attitude toward the implementation of this tool. Most GPs acknowledged that because they do not see a large proportion of pediatric patients in their day-to-day practice, they might lose proficiency in treating these patients. As a result, they welcomed DIRECT-T1DM as a learning opportunity. Younger doctors recognized that in their teaching practices, there is an emphasis on evidence-based, guideline-driven tools for learning. Hence, they acknowledged that their practices may be more open to DIRECT-T1DM in comparison to other nonteaching practices that may not have such a strong emphasis on learning.

#### IT Infrastructure

DIRECT-T1DM requires the installation of FHT clinical decision support software. Not all GPs interviewed practiced in clinics with FHT installed. GPs without adequate IT infrastructure reflected that it may be harder to convince practices to install CDSS that sits outside their EMR system than to adopt the DIRECT-T1DM intervention. Therefore, this is a barrier that would decrease the use of DIRECT-T1DM. Some GPs mentioned that DIRECT-T1DM was similar to adverse drug interaction alerts that were already embedded within their EMRs (without the use of FHT), and they believed that DIRECT-T1DM could be implemented into community general practice this way. For example, GPs saw this support tool as being a potential integration within EMR Software Best Practice, which is used by 65.8% of Australian general practices [22].

### Outer Setting Domain

#### Overview

The outer setting in CFIR refers to the broader context in which the inner setting resides [17], which, in this case, is the broader health care system. DIRECT-T1DM could be optimized to fit considerations that exist due to the outer setting. Table 5 summarizes the additional considerations due to external pressure in the outer setting.

**Table 5:** External pressures in the outer setting.

Construct and key themes		Representative quotes
**External pressure**		
	Overreferral to the emergency department	“I guess there’s a risk that, and maybe an acceptable risk, that you over refer to ED, and that you’re sending people to ED that just have a viral illness...So yeah, I guess that’s a real, it’s a consideration right now. It is so swamped.” [GP^a^3] “Yeah, I’m probably just going to send them to emergency, but this is kind of good, I think, to back up your decision making or if you’re not quite sure you did the right thing.” [GP5]
	Accessibility of pediatric specialists	“I guess this is worth bringing to people’s attention. Just to discuss with the paediatric specialists. However, in my current clinic access to paediatric specialist is really challenging. For a variety of reasons: wait times, financial barriers. Yeah. So, I feel like that’s good, that’s nice, yeah. But in reality, as a GP, you gotta do something while [the patient’s] waiting to be seen.” [GP3]
	Societal stigma surrounding diabetes	“Definitely, if you come in, and...you’ve got some something on the screens telling you’ve got diabetes. Yeah, you’d be pretty distressed. And I think that there’s a lot of stigma associated with diabetes. And if you’re getting that as a child, it’s amplified.” [GP3] “I think it would take a bit of explanation to try and give the parent the idea that there’s a couple of types of diabetes, type 1 diabetes, yes, they need regular insulin. But if they don’t get their insulin, they get very sick, very quick. Yeah, so, you’d have to sort of really, you know, explain, or emphasize the need for urgent care.” [GP1]

^a^GP: general practitioner.

#### External Pressure: Secondary and Tertiary Care Considerations

Some GPs felt that overreferring to the ED was a consideration when assessing children with potential T1D. GPs were conscious of the exacerbated wait times that exist within EDs currently; however, they believe that the risk of overreferral was an acceptable risk given the consequences of undiagnosed T1D. DIRECT-T1DM was helpful in reinforcing and encouraging the clinical decision to refer to the ED. When T1D was suspected, GPs found the clinical decision support recommendations to be thorough, including clear instructions to refer the patient to the nearest ED. This is because not all practices will have access to pediatric endocrinologists, which, for some, is related to the location of practice in relation to tertiary settings. Instead, such practices would refer the child to the nearest ED where they are also equipped to treat DKA. In contrast, participants identified that the clinical decision support recommendations for treatment of type 2 diabetes may not be inclusive of all practices where access to pediatricians for the treatment of type 2 diabetes may be limited due to wait times and financial barriers. GPs recommended tailoring the information in this section of the pop-up.

#### External Pressure: Societal Stigma

Societal stigma surrounding diabetes and preconceived expectations regarding the consultation may play a role in how the instructions in the DIRECT-T1DM intervention are communicated. Parents or patients may not expect that they will be referred to the ED following a visit to the GP for relatively nonspecific symptoms.

Multimedia Appendix 3 summarizes how the context provided in the interviews shaped design recommendations pertaining to DIRECT-T1DM.

## Discussion

### Principal Findings

Major themes were identified within 7 constructs across 4 CFIR domains: outer setting, implementation process, inner setting, and innovation. Acceptability of the initial DIRECT-T1DM clinical prompt and recommendations (when T1D was suspected) was strong because of its straightforward design, the fact that GPs did not anticipate seeing it often, and the severity of the consequences associated with DKA. Design recommendations were driven primarily by the desire for information to be highly specific to management at the point of care as well as to adjust the design packaging to reflect the urgency of the prompt. Acceptability of recommendations in DIRECT-T1DM when the GP did not suspect T1D was mixed: some GPs found that the information provided was not specific enough to children, others appreciated the reminder regarding the risk of T1D despite BMI status. One GP found this recommendation not to be useful and would ignore it in practice.

### Scarcity of Time in General Practice Shapes Design Needs for Clinical Decision Support

Prior research has demonstrated that facilitating GP needs within the context of the inner and outer settings is beneficial to intervention adoption [23]. Having a short amount of available time in general practice is commonly reported among interviewees, not only in our study, but across the country [24]. This influences the amount of time that GPs would like to spend using a clinical decision support tool, emphasizing the need to tailor it for usability at the point of care. Secondary care considerations of ED overreferral and patient perception have, to our knowledge, not been explored within the research involving early T1D recognition. However, these issues have been explored in the broader general practice context, where concerns about overreferral [25], the tool’s influence on patient perceptions, and its impact on communication have been documented [26]. Our study supports that design considerations should involve appraising the time spent understanding and deciding about the information within the tool.

### Situating T1D Within a GP’s Caseload

Difficulty in diagnosing pediatric T1D stemmed from a combination of the lack of available time in consultation to consider it as a possibility, nonspecific presentations, and the relative scarcity of children with T1D in the population. This is consistent with existing qualitative interviews assessing the challenges in receiving a T1D diagnosis [5,27,28]. The influence of new-onset T1D on a GP’s workload was exemplified in our study, as in a combined approximately 80 years of GP experience, only 1 patient was diagnosed with new-onset T1D. This is different from existing qualitative interviews, which targeted GPs who had previously diagnosed children with new-onset T1D [5,28]. This informs an existing research gap, as the perceptions of GPs who have not previously seen new-onset T1D in practice may be more reflective of most GPs’ experiences and attitudes when encountering a clinical decision support tool, such as DIRECT-T1DM. Including GPs without prior experience of diagnosing T1D at new onset increases the representativeness of GPs’ perceptions of the challenges and barriers to diagnosing T1D and may demonstrate the differences in perceptions of how a child may present at new onset when compared to GPs who have diagnosed T1D in the past.

### Pathology Testing Is Conducted at a Lower Level of Suspicion, Which May Be Common Among Patients With New-Onset Type 1 Diabetes

Existing research studies assessing pathology referrals from general practice are quantitative in nature [6,9,29,30]. Our study provides clinical context as to why this may be occurring in general practice. We found that the burden of time pressure may lead GPs to prefer laboratory testing over point-of-care testing. In addition, GPs may have elected to conduct the pathology test instead of point-of-care testing in cases where their suspicion of T1D is low, as part of a broader panel of tests to narrow down the child’s diagnosis. Prior qualitative interviews with GPs who have diagnosed a child with new-onset T1D have demonstrated that often, the child may present “well” and not at a level of severity that would induce suspicion of T1D or evoke a sense of urgency to refer to the ED [5]. Combining our findings with this existing research may demonstrate that pathology tests that delay diagnosis of T1D may be requested in general practice because symptom presentation is not specific enough to diabetes or severe enough to evoke a sense of urgency from the GP’s perspective. This, in turn, modifies our design considerations for the tool, embedding language that encourages GPs to exclude pediatric T1D as a possibility, even at a lower level of suspicion.

### Integration With Clinical Decision Support Tools at the Appropriate Time Within a GP’s Workflow Is Critical to Acceptability

Acceptability of DIRECT-T1DM as a concept was high. This was because of its capacity to increase patient safety and because GPs did not anticipate encountering the tool often. This is in line with existing research evaluating clinical decision support tools, as the timing of advice is important to whether GPs engage with them in practice [14,31]. Design considerations provided by GPs were intended to improve the ease of use, fit into workflow, increase the involvement of patients, and improve the presentation and specificity of advice. Existing research has demonstrated that these are important facets of innovation design, particularly with clinical decision support tools, influencing the degree to which they are used in practice [32].

### Strengths and Weaknesses of This Study

The think-aloud protocol was advantageous as it allowed for the recognition of instantaneous response to the tool. A lot of the feedback about design packaging, relative priority, and usefulness of the tool was collected upon on-the-spot interaction with DIRECT-T1DM. Participants then had a chance to elaborate on their feedback in the postsimulation interviews. This is in line with what previous comparisons between “think-aloud” and “near-live” simulation studies have identified [16]. However, it has also been identified that “think-aloud” protocols are not as beneficial when compared to “near-live” protocols when assessing compatibility with workflow and relative priority [16]. As a result, our conclusions regarding workflow compatibility and priority may be weakened. Future simulation studies involving GPs can consider the “think-aloud” protocol as a tool for evaluating clinical decision support tools, especially in cases where it is unfeasible to adopt a “near-live setting.” Times where GPs can pause and take a break should be considered within the interview design, as the “think-aloud” process may induce a higher than usual level of burden on the participant.

We elected to recruit a small sample of GPs (N=6). This sample size may pose limitations toward the application of our conclusions to the wider population. Furthermore, the interviewer (CB) had preexisting professional relationships with 3 of the GPs taking part in the simulations before the initiation of the study. One GP had collaborated on a different study involving DKA prevention research. We recognize that this may skew perceptions favorably toward DIRECT-T1DM and that there is limited generalizability of our findings as a result. Further investigations following implementation within the community involving GPs, such as qualitative interviews and surveying, will be conducted to assess the acceptability of this tool with a larger sample of clinics. Our study aim was to evaluate the usability and acceptability of DIRECT-T1DM, rather than to assess GP performance when encountering patient scenarios that involve possible T1D diagnoses. Therefore, we cannot draw conclusions regarding the awareness of best practice guidelines or GP performance in such scenarios. In the future, we plan to investigate whether the tool effectively optimizes the management of suspected T1D in the community.

Adoption of CFIR [17] allowed for in-depth analysis of the effect of different settings, contexts, and GP needs on design considerations and optimizations for our tool. We were able to identify critical clinical context surrounding the challenges in diagnosing T1D and how we can improve our tool to meet the needs that arise because of these challenges. We also identified a need for embedded risk prediction tools in general practice for the development of pediatric T1D to respond to the challenge of recognizing T1D in children with nonspecific symptoms in busy general practice.

### Implications for Future Research

This study informs the optimization of DIRECT-T1DM and suggests that there is possible clinical utility as well as end-user acceptability. As a result of this investigation, changes will be made to DIRECT-T1DM, including the use of more alarming colors, specific language and guidelines, and the development of a patient information handout. We anticipate that these optimizations to the tool will enhance its usability and acceptability. DIRECT-T1DM could also be integrated into EMR systems, such as Best Practice, rather than being embedded within FHT, and this important preliminary work informs the integration of our tool, ensuring that it is acceptable and useful before broader implementation.

Once DIRECT-T1DM is implemented in the community settings, further evaluation will take place for all stakeholders. Qualitative interviewing as well as surveying of GPs using the tool will be undertaken to ensure that the tool is usable and acceptable. Auditing the rate of pathology test requests in general practice, as opposed to point-of-care testing that is taking place, will also be undertaken. Rates of DKA and referral following a point-of-care testing can be measured by our local EDs at the Royal Children’s Hospital, and retrospective identification of patients and their caregivers that were referred to the ED because of our tool can be interviewed for insights into their experiences.

All GPs within our study identified a need for an additional clinical decision support tool for the context of new-onset pediatric T1D where early symptoms are nonspecific and may be underappreciated by the GP and caregivers alike. It was noted in this study and in the existing literature that usually T1D-related symptoms are not deemed serious enough by the patient to be reported as the presenting complaint when attending general practice [5,27,28]. As DIRECT-T1DM is triggered upon request of a diabetes-related pathology test, it will not address cases where T1D is not “front of mind” and, therefore, not address the cases where T1D is misdiagnosed. Future research can consider the development of risk prediction tools for the purposes of alerting GPs when a child is at elevated risk of developing T1D before the consultation. Our investigation also demonstrates that a “think-aloud” protocol is suitable for qualitative evaluation of CDSS tools when it is unfeasible to adopt a near-live simulated protocol.

## Appendix

Multimedia Appendix 1 COREQ (Consolidated Criteria for Reporting Qualitative Research) checklist.

Multimedia Appendix 2 Interview script, including the clinical scenarios.

Multimedia Appendix 3 Summary of the relationship between the outer setting, implementation process domain, and innovation domain.

## Abbreviations

CDSS: clinical decision support system
CFIR: Consolidated Framework for Implementation Research
DIRECT-T1DM: Decision-Support for Integrated, Real-Time Evaluation and Clinical Treatment of Type 1 Diabetes Mellitus
DKA: diabetic ketoacidosis
ED: emergency department
EMR: electronic medical record
FHT: Future Health Today
GP: general practitioner
T1D: type 1 diabetes
