# Supplementary material for: Exploring the User Acceptability and Feasibility of a Clinical Decision Support Tool Designed to Facilitate Timely Diagnosis of New-Onset Type 1 Diabetes in Children: Qualitative Interview Study Among General Practitioners
Source: JMIR Form Res. 2024 Sep 23;8:e60411. doi: 10.2196/60411 (PMC11459099; doi:10.2196/60411)
Supplement: Multimedia Appendix 2 [file formative_v8i1e60411_app2.docx]

Thank you for agreeing to be part of our simulation study evaluating our decision support tool for type 1 diabetes in general practice.

**PLS/CONSENT FORM:** Before I get into the actual study, I just wanted to make sure that you are still comfortable to go ahead and are happy with the contents of the PLS and consent form. If you haven’t signed it, I have a copy here for you to read through and sign whenever you are comfortable. If you have any questions, don’t hesitate to ask. When you are ready, we will start today by asking you a few questions. You will be recorded here.

**INTRO:** We’d like to start off by asking you a few general questions about your experience as a GP, as well as use of tools like Future Health Today etc. To start off with, I would like to gain your consent that you are happy for me to record this initial interview before we start the simulation.

Wonderful, I’ll now start off with the questions:

**Pre-sim interview questions:**

**We will start off with a few questions about your experience with the technology being used here.**

1. Are you aware of **FHT**?
   1. How did you become aware of FHT?
   2. Do you use it in your practice?
   3. How is it used in your practice?
2. Have you used **best practice** EMR before? and whether they need any coaching through the different EMR?

**We will now move to your experience as a GP**

How long have you been practicing as a GP?

Where is your practice/s located?

**Now we will talk more specifically about paediatric diabetes in primary care.**

Retrospective hospital audits have shown that a diagnostic delay for paediatric type 1 diabetes is associated with increased DKA severity and frequency. A delay of 48 hours is associated with a twofold risk of deterioration to DKA.

It has been found that there are several reasons for delay, there may have been a delay in recognition of symptoms from the child and/or their parents, there could have been a delay in securing an appointment with a GP, and there are also delays from after the GP has been visited. The two most common delays after a GP have been visited include misdiagnosis and awaiting the results of blood tests from laboratories.

My study is looking at implementing a clinical decision support tool to address this last aspect to delay, it is intended to promote use of point of care tests and urgent referral to emergency departments instead of ordering and awaiting the results of diabetes blood tests.

The clinical decision support tool is essentially a real-time alert that will appear when a diabetes laboratory blood test is ordered for a child who is under 18, who does not have a diagnosis of diabetes or PCOS recorded.

Before we move to the sim, I would like to ask you a few questions about your experiences with diabetes and PCOS.

Type 1 diabetes is relatively rare in the population, have you had any experience with diagnosing type 1 diabetes in a child?

If you have had experience in diagnosing type 1 diabetes, can you tell me more about your experiences with this?

What are some of the challenges with making this diagnosis?

What would have been helpful for you in these cases?

We have found that there has been an increase in screening for type 2 diabetes in general practice in Victoria, have you had any experience with young people presenting with possible type 2 diabetes?

How would you differentiate here?

Are you aware of any **clinical practice guidelines** which guide the diagnosis and management of new onset diabetes in young people?

Are **you aware of any reported data** about how GPs manage type 1 diabetes at its onset?

We have also found that there may be increased screening for PCOS in adolescence, have you had any experience with this?

Do you have any further comments you would like to make about this before we start?

**EXPLANATION ABOUT SIM:**

**CAMERAS, LOGISTICS:**

Great, thank you for answering those questions. We will move on to the main part of the study now, the simulation. We will be using the SimLab located here that is set up like a clinic. This room is visually, and audio recorded, with cameras here and here.

The screen is also recorded as you interact with it. There is a double-sided mirror there where my team will be observing just to make sure everything is running smoothly.

**AIM OF STUDY:**

The intention of this study is to test whether our decision support tool for type 1 diabetes is feasible to be used in practice, whether it is acceptable to the user (you) and to evaluate our tool for any improvements that you might suggest.

As I mentioned, our clinical decision support tool is designed to pop-up when a diabetes related pathology blood test referral is ordered for a child or young person, under 18, who is not already diagnosed with diabetes or PCOS.

The pop-up is designed to prompt immediate referral to the nearest emergency department instead of awaiting the results of laboratory tests, in line with RACGP best practice guidelines.

**SCENARIOS:**

I have designed, in consultation with a few GPs and a paediatric endocrinologist, five scenarios where a blood glucose test could be ordered for a child or young person, under 18 years of age. Not all these scenarios were written specifically for type 1 diabetes.

I will present you with three of these scenarios. We don’t have actors for this simulation, I will come in and talk to you about each patient scenario.

Although I am not acting, I would like you to take on the persona of a GP who is not thinking about type 1 diabetes and respond to these situations as I present them to you.

In all cases, I will try to steer you to order the diabetes blood test you think is suitable. Even if, in line with guidelines that I have mentioned before, direct referral is the better option.

**THINK ALOUD:**

I’d like to state that we are not testing you as a GP in any way, rather we are looking for your perceptions about using the tool in different clinical scenarios. This includes in situations not related to diabetes, where the pop-up may also appear. Do you have any questions so far?

Fantastic. We will also employ a think aloud protocol throughout the simulation, so I want you to be as verbose as possible. Even if it sounds silly, I want a window into your mind as you interact with the pop-up. Use as many words as you possibly can to describe what you are thinking and doing.

The software we have currently designed this tool for is Best Practice, are you familiar with this software or will you need coaching?

**SCENARIOS – START:**

Great, let’s get started with the first scenario then. Feel free to ask me any questions about the patient throughout the simulation if you feel you need more information. Once you feel you have completed using the tool for the first patient, and have no other immediate feedback to give, let me know, and we will move on to the next one. I have a notepad and pen here, if you would like to make any notes about the patient. Feel free to also make these notes in the notes section of BP.

**Scenario #1 [T1DM onset – adolescence]**

Jason, a 15-year-old patient presents with his mother, Carmen, to your clinic with symptoms of fatigue and unexplained weight loss. This was noticed as his school uniform is now too big for him. He also expresses that he cannot stop feeling thirsty even after he has something to drink. He explains that his tiredness and excessive thirst began around a week ago. Today, he vomited a few times.

Jason and his family have moved to Parkville from interstate for his parents’ new job. Your clinic is relatively new to him, only having consulted with Jason once before: 3 weeks ago. This was for an upper respiratory tract infection. The GP who treated him, Kimberlee is on maternity leave, and they have booked an appointment with you for this appointment.

**Scenario #2 [T1DM onset – under five years old]**

Gisele, a 2-year-old patient presents with her mother, Carmen, to your clinic. Carmen explains to you that her daughter was vomiting excessively last night, and that for the past few days she has looked very tired and lethargic. She has been crying all day and has wet the bed a few times in the past few weeks despite not having done so for a while.

She has been a patient at the clinic since she was born. Her mother, Carmen, has booked in for you to see her daughter today.

**Scenario #3 [Suspected PCOS]**

Taylor, a 17-year-old patient presents to your clinic. She is concerned because her periods have started to become irregular the past few months. Last month, it lasted two weeks and then she didn’t have a period when she expected to for her next cycle. She has been feeling fatigued lately and has noticed that she has gained weight. She has also noticed that her skin has started to break out with acne. She has tried to participate in more sports at school, and change her skincare routine, but nothing is seeming like it helps. Her family recently moved house, and she has come to you to be her new treating GP.

**Scenario #4 [Possible T2DM]**

Damien, 15, presents with his father, Michael, to your clinic. Michael is concerned as his son has gained a lot of weight in the past year. Damien informs you that he has been feeling very fatigued lately and has a lot of trouble motivating himself to do daily tasks. Michael is worried about his son because he is a type 2 diabetic, and he knows that this puts his child at higher risk.

Damien has only had three visits at your clinic before, once for an upper respiratory tract infection, once for a general check-up, and once for immunisations.

**Scenario #5 [T1DM – parental concern]**

Kayla, 7, presents with her mother, Kelly, to your clinic. Kelly is a type 1 diabetic and is worried that her daughter may develop it in the future. Neither Kayla, nor her mother, Kelly, reported any symptoms suggestive of type 1 diabetes in this visit. However, Kelly would still like this to be investigated.

**Post sim:**

Thank you so much for your contribution in the simulation part.

Let’s take a few minutes to have a break and get some water, stretch our legs, etc. We will reconvene in the sim room to do the post-sim interview.

The purpose of this next interview is to evaluate the pop-up. I would like your consent again that you are happy for me to record this next part.

**Feedback:**

To start off with, do you have any thoughts/feedback/comments about the process of this sim for me to make it better for the next GP?

**Intervention – overall:**

Do you think that there is a need for this intervention? Why/Why not?

Did you think that the pop-up was useful? Why/Why not?

What did you like about the tool?

What did you not like about the tool?

How complicated or uncomplicated was the tool?

**In your practice:** Now I would like you to think about your own practice.

In the real world, do you think this pop-up would be effective for its purpose? Why/Why not?

How does this alert compare with other alerts in your practice?

What are its pros and cons compared to existing programs?

If you are using FHT, how does this pop-up compare?

Think about the other GPs in your practice, do you think that they would use this?

How well do you think the pop-up fits within the existing workflow of your practice?

Who would be involved in enacting the instructions for this pop-up from start to end?

Do you think any changes would need to happen in your practice to accommodate this pop-up?

Is there anything we would need to change or keep about the intervention to make it suit your practice?

Do you think your practice is ready for this pop-up? If we had the prototype ready today, would your practice be ready for it?

Are you confident that at your practice you would be able to successfully implement the intervention?

Do you think there would be any barriers or gatekeepers in your practice that would stop the implementation of this tool from being a success?

**Patients:** Now let’s talk about your patients

How do you think your patients would react if, after presenting with relatively non-specific symptoms, were instructed to:

Complete a point of care (finger-stick, urine) test?

Go straight to the ED?

Would you use this tool to explain to the patient the need for the point of care tests or the hospital visit?

If your patient saw this on screen, how do you think they would respond?

**Pop-up:** Now let's talk about the pop-up itself

What do you think about this initial box here? Was its place useful?

Let's talk about what happens when you answer yes – do you think that there is too much here, not enough?

Another GP I spoke to in the initial design of the prototype thought that there should be boxes with symptoms and classification for the blood glucose levels, do you think something like this would be helpful or would take away from the tool?

Let's talk about the links we have added – were you aware that these were links or was that not obvious enough?

The links take you to guidelines about the management of paediatric diabetes – are these links sufficient or would you have liked more resources?

Were you aware of some of these new guidelines?

Do you think this is the appropriate intervention to address the problem I have outlined?

Why/Why not?

If another is better, can you describe what an alternative suggestion might be?

Why would clinicians prefer the alternative as opposed to this one?
