# Supplementary material for: Exploring the User Acceptability and Feasibility of a Clinical Decision Support Tool Designed to Facilitate Timely Diagnosis of New-Onset Type 1 Diabetes in Children: Qualitative Interview Study Among General Practitioners
Source: JMIR Form Res. 2024 Sep 23;8:e60411. doi: 10.2196/60411 (PMC11459099; doi:10.2196/60411)
Supplement: Multimedia Appendix 3 [file formative_v8i1e60411_app3.docx]

| **Context provided in interviews:** | **DIRECT-T1DM design recommendations**  **(INNOVATION DOMAIN)** |
| --- | --- |
| Alert fatigue: Pop-ups are easy to disregard. | Advantage of DIRECT-T1DM’s central design. Further recommendations were made to increase how alarmist our pop-up was (add red font/colouring, add exclamation marks, add capital letters) |
| Cognitive load and time pressure: Too much information to appraise to make a decision within the timeframe available. | DIRECT-T1DM has the key information, it should be emphasised further, with links and other information still there, but minimised. |
| Workflow compatibility: Time availability may play a role in referring the child for external laboratory testing rather than conducting point of care testing. | Further recommendations to increase relative priority of DIRECT-T1DM: alarmist colours, bold font, emphasis on prevention of deterioration to DKA. |
| Relative advantage: Time availability increases difficulty to recognise when a patient is presenting with T1DM. | Out of scope for DIRECT-T1DM. Further support is required to address this issue. |
| Relative advantage: Symptoms are non-specific, easily attributable to other, more common illnesses. Safety netting: what happens when you don’t even think of T1D as a possible diagnosis? | DIRECT-T1DM is triggered following initial suspicion of T1D, as a diabetes related pathology test is required. Consideration of a complementary decision support tool addressing these concerns would be beneficial. |
| Relative priority: ‘Grey area’ cases, pathology testing for T1D is likely at a low level of suspicion | DIRECT-T1DM can improve in communicating the importance of conducting point of care tests, even at a lower-level suspicion of T1D. This includes suggested changes to language, colour of the pop-up, and restructuring of the information in the part of the alert that occurs when GPs suspect T1D. |
| External pressure: ‘Swamped’ emergency departments: GP concerns regarding over-referral | DIRECT-T1DM’s inclusion of links to guidelines from sources with strong evidence bases (e.g. RACGP) aids in affirming the decision to refer. |
| External pressure: Paediatric specialists may not be accessible for all clinics in the treatment of adolescent type 2 diabetes | DIRECT-T1DM should include more direct and focused information for the screening, diagnosis, and management for adolescent type 2 diabetes and/or polycystic ovary syndrome. |
| Consumer information: Patients and caregivers may require additional information about diabetes and future management while they are transferred to emergency. | A plain language statement, or information sheet for patients and their caregivers should be included within DIRECT-T1DM, to provide for families where hyperglycaemia is confirmed, and they are sent to the emergency department. |
